# Development and psychometric evaluation of a measure to evaluate the quality of integrated care: the Patient Assessment of Integrated Elderly Care

**DOI:** 10.1111/hex.12391

**Published:** 2015-07-31

**Authors:** Ronald J. Uittenbroek, Sijmen A. Reijneveld, Roy E. Stewart, Sophie L.W. Spoorenberg, Hubertus P.H. Kremer, Klaske Wynia

**Affiliations:** ^1^ Department of Health Sciences, Community and Occupational MedicineUniversity Medical Centre GroningenUniversity of GroningenGroningenThe Netherlands; ^2^ Department of NeurologyUniversity Medical Centre GroningenUniversity of GroningenGroningenThe Netherlands

**Keywords:** Chronic Care Model, elderly people, patient assessment, psychometric properties, quality of care

## Abstract

**Background:**

Novel population‐based integrated care services are being developed to adequately serve the growing number of elderly people. Suitable, reliable and valid measurement instruments are needed to evaluate the quality of care delivered.

**Objective:**

To develop a measure to evaluate the quality of integrated care from the perspective of elderly people, the Patient Assessment of Integrated Elderly Care (PAIEC), and then to assess its psychometric properties.

**Methods/Design:**

After the Patient Assessment of Chronic Illness Care was adapted to the PAIEC, a cross‐sectional postal‐survey study was performed among 223 elderly people who received integrated elderly care and support. We assessed the factor structure, internal consistency, known groups and divergent validity using robust nonparametric tests.

**Results:**

Mean age of participants was 83 years (standard deviation 4.7), and 69% was female. The original five‐factor model was rejected; a good fit was found for a three‐factor model, when excluding the item on patients' satisfaction with care. The PAIEC and its subscales showed good internal consistency (ordinal alphas > 0.90). Known‐groups validity was supported regarding number of medications, prevalence of chronic conditions and home care received. No differences were found between groups based on sociodemographic aspects. Divergent validity was supported by low correlations (Spearman's rank correlation coefficients < 0.30) between PAIEC scales and measures of quality of life, complexity of care needs and frailty.

**Conclusion:**

The PAIEC seems to have considerable potential as a reliable and valid measurement instrument that evaluates quality of integrated care and support from the perspective of elderly people.

## Introduction

As a result of changing patterns in the demand for health care, health‐care systems are being compelled to embrace person‐centred and integrated care services.^1^, ^2^ These still evolving services enable health‐care systems to provide a continuum of modern self‐management support, and age specific, coherent, proactive, and preventive care and support. Person‐centred and integrated care services are based on the needs and expectations of persons and their informal network, and not only on diseases.^1^ For the development of such integrated care services, the Chronic Care Model (CCM) provides an internationally accepted and evidence‐based framework.^3^ Novel population‐based integrated care services for elderly people based on or related to the CCM have been introduced. Examples include the Program of All‐inclusive Care for the Elderly,^4^ Guided Care^5^ and Embrace.^6^ It is of importance to evaluate the quality of care delivered within these new services and essential to incorporate the patient's perspective.^6^, ^7^, ^8^, ^9^

The Patient Assessment of Chronic Illness Care (PACIC) is a measurement instrument that evaluates the quality of CCM‐based chronic illness care from the patient's perspective.^10^, ^11^ It was developed and validated for patients with a chronic condition, for example diabetes and chronic heart failure,^12^, ^13^ and has been translated into numerous languages. A PACIC version which reflects the care and support for elderly people with a great life‐course diversity regarding multiple chronic conditions and age‐dependent disabilities is currently not available. Such a version should avoid questions related to diseases only and focus on age‐appropriate integrated care and support.^14^, ^15^

The aim of this study was to develop a measure to evaluate the quality of integrated care from the perspective of elderly people, the Patient Assessment Integrated Elderly Care (PAIEC) and then to assess its psychometric properties, taking the PACIC as starting point.

## Methods

### Design, setting and procedure

A cross‐sectional postal‐survey study was performed. Data were collected on elderly people, 75 years and older, who had participated in the Embrace study, a randomized controlled trial, to examine the effectiveness of a new CCM‐based intervention among Dutch community‐living elderly people. In the source study, the response was 49.7%. In total, 223 elderly people who were identified as frail or having complex care needs at baseline, and who had received a year's integrated care and support under the aegis of Embrace, were selected for this study (for a detailed description of the Embrace study, see Spoorenberg *et al*.).^6^

All participants provided written consent after being informed about the content of the Embrace study and the consequences of involvement. Data were collected using self‐reported questionnaires. The PAIEC was part of a more extensive questionnaire. This questionnaire was divided into a number of sections. Participants were advised to take a break after every section and were offered support in filling out the questionnaire, that is a volunteer was available via the project helpdesk. For our present study, the 12‐month follow‐up measurement was used. The Medical Ethics Committee of the University Medical Center Groningen assessed the Embrace study proposal and concluded that approval was not required (Reference METc2011.108).

### Intervention

Embrace (in Dutch: ‘SamenOud’) is a person‐centred, integrated care service for community‐living elderly people. Embrace combines the CCM with use of three risk profiles (Robust, Frail and Complex care needs) based on the Kaiser Permanente (KP) Triangle. The profile ‘Robust’ includes non‐frail elderly persons without complex care needs. The profile ‘Frail’ includes frail elderly who are at risk of developing complex care needs. The profile ‘Complex care needs’ includes frail elderly people with complex care needs. All these elderly people received integrated care and support, but with differences regarding number of contacts, main focus, that is either on health‐related or social problems and individual vs. group approach.

Multidisciplinary Elderly Care Teams – each consisting of a general practitioner, an elderly care physician and two case managers (district nurse and social worker) – provided coherent, individualized, proactive and preventive care and support. Elderly persons within the ‘Frail’ and ‘Complex care needs’ profiles received individual support from a case manager. Case managers frequently visited these elderly persons and assessed their situation, created in co‐operation with the elderly person an individual care and support plan, implemented this plan, monitored the situation and navigated the realization of this plan. During monthly meetings, the Elderly Care Team discussed and evaluated the health status and social situation of their clients. If necessary, they act proactively to prevent downfalls.

### Measurement instruments

The PAIEC is an adapted version of the PACIC measurement instrument. The original PACIC comprises 20 items, which were aggregated into five *a priori* defined CCM‐based subscales: ‘Patient Activation, Delivery System Design/Decision Support, Goal Setting/Tailoring, Problem‐Solving/Contextual and Follow‐Up/Coordination’.^12^ Respondents rate how often they perceived the care and support as described in each item during the past 6 months. Response options range from never (1) to always (5).^12^

The PACIC was adapted in three steps to create the PAIEC. First, the Dutch version of the PACIC for diabetes and COPD^16^ was adapted by Dutch researchers so that it would be applicable to the population of elderly people (RJU, SLWS and KW). The researchers are experts on aging, elderly care and quality of care, and have the command of the Dutch and English languages at academic level. Most noteworthy of the adaptations were those related to the concepts ‘chronic condition’ and ‘treatment’, which were converted into ‘consequences of ageing’ and ‘care and support’, respectively. One item was added: ‘I was asked whether I had any problems with care and support or about my experiences with either’. Furthermore, as not all elderly people receive the same intensity of care and support, the response option ‘does not apply’ was added to prevent missing values. To gain a more realistic estimation of the receipt of integrated care, scale sum scores were calculated after the response option ‘does not apply’ and missing values were recoded into ‘never’. Second, the feasibility of the preliminary PAIEC was pre‐tested for clarity, comprehensiveness, redundancy and patient burden in a random sample of eight community‐living older adults (five women and three men, aged 61–84 years); no additional modifications proved to be needed. Third, the Dutch version of the PAIEC was back‐translated by two native English speakers. Discrepancies were discussed by three researchers (RJU, SLWS and KW), resulting in a version of the PAIEC that comprised 21 items and that could be used for psychometric evaluation.

The complexity of care needs was assessed by means of the INTERMED Elderly Self‐Assessment (IM‐E‐SA) measurement instrument.^17^ The IM‐E‐SA consists of 20 items in biological, psychological, social and health‐care domains. Questions and ratings per domain are related to three time periods: past, present and future. Scores are summed, and the total score ranges from 0 to 60; the higher the score, the higher the level of complexity of care needs. The internal consistency was satisfactory with a Cronbach's alpha of 0.78 in a previous study among elderly persons^17^ and 0.73 in this study.

Frailty was measured using the Groningen Frailty Indicator (GFI).^18^ The GFI is a self‐report measurement instrument that ‘assesses frailty in the physical, social, cognitive and psychological domains’.^18^ Scores on the 15 items are summed where a higher score (0–15) indicates a higher level of frailty. The internal consistency was acceptable with a Cronbach's alpha (KR20) of 0.68 in a previous study among elderly persons^18^ and 0.60 in this study.

Life satisfaction was evaluated using the Cantril's Self‐Anchoring Ladder of Life scale.^19^ The response options range from 0 to 10, where a higher score indicates better life satisfaction.

Health‐related quality of life was evaluated by means of the EuroQol 5D‐5L (EQ‐5D‐5L).^20^ This measurement instrument consists of five items that reflect on five domains: mobility, self‐care, pain, usual activities and psychological status. An index score (0–1) was calculated where a higher score indicates a better health status. The internal consistency of the EQ5D‐5L was good in a study among HIV patients: Cronbach's alpha was 0.85^21^ and 0.75 in this study. The EQ‐5D‐5L also contains a standard visual analogue scale (VAS) for assessment of an individual's rating of his/her current health status. The VAS ranges from 0 to 100; a higher score indicating a better state of health.

Finally, participants were questioned about the following demographic and health‐related characteristics: age, gender, educational level, marital status, number of chronic conditions, number of medications and home care received.

### Data analysis

Demographic, health‐related characteristics and data quality were analysed using descriptive statistics. The psychometric properties of the PAIEC were examined by assessing the factor structure, internal consistency and construct validity of the scales.

Confirmatory factor analysis (CFA) was applied to examine the *a priori defined* five‐factor structure.^22^ Before factor analysis, the response option ‘does not apply’ was recoded into the response option ‘never’. Next, items were analysed as ordinal variables with a robust weighted least‐square method estimator.^23^, ^24^ The goodness‐of‐fit was assessed by means of a combination of absolute goodness‐of‐fit statistics [root mean square error of approximation (RMSEA), weighted root mean square residual (WRMR), standardized root mean square residual (SRMR)] and incremental fit indices [comparative fit index (CFI) and Tucker–Lewis index (TLI)]. The model was considered to have a good fit if the RMSEA ≤ 0.06, WRMR ≤ 1.00, SRMR ≤ 0.08, and CFI and TLI ≥ 0.95.^25^ Exploratory factor analyses (EFA) were performed if the *a priori defined* five‐factor model was rejected. To investigate an alternate structure for this data, the items were analysed as ordinal variables with a robust weighted least‐square method estimator and using oblique rotation.^26^ An EFA factor structure was accepted if item regression coefficients were >0.40, items predominantly loaded on one factor only, the estimated error variances were positive, and criteria for the goodness‐of‐fit indices as described previously were met.

Internal consistency of the PAIEC total scale and subscales was evaluated by calculating the ordinal alpha. Alpha ≥0.70 was defined as optimal.^27^ Subsequently, scales were constructed and scale scores were summed.

Known‐groups validity was examined using the Mann–Whitney and Kruskal–Wallis tests. Based on the expected relationships as stated earlier by Glasgow *et al*.,^12^ it was hypothesized that the PAIEC scales would not discriminate statistically significantly between subgroups of respondents based on differences in gender, age (two groups: older or younger than mean age of the sample), marital status (two groups: married or living together; and single, divorced or widowed) and educational level (three groups: low, moderate and high). In addition, we expected no differences between the frail participants and those with complex care needs as elderly persons in both profiles received the same degree of integrated care and support. On the other hand, we hypothesized that elderly persons who are supposed to need more care and support will receive a higher intensity of integrated elderly care. Therefore, PAIEC scales should be able to discriminate statistically significantly and clinically relevantly between subgroups of respondents known to differ on relevant clinical characteristics: number of chronic conditions (3 or less vs. 4 or more conditions), number of medications (3 or less, vs. 4 or more medicines)^12^ and receiving home care (yes vs. no). The effect size for nonparametric tests (coefficient *r*) for unrelated samples was calculated for statistically significant group differences^28^ with a coefficient *r *≥* *0.10 reflecting a clinically relevant difference between groups.^29^

To test whether PAIEC exclusively measures the constructs of a patient's perception of care and support as experienced, the divergent validity of the PAIEC was examined by calculating Spearman's rank correlations. It was hypothesized that correlations between the PAIEC variables and discrim‐inating variables for complexity of care needs, frailty, life satisfaction and health‐related quality of life would be weak (<0.30).

Statistical analyses were conducted using SPSS/PASW 20 (IBM Corp. Released 2011. IBM SPSS Statistics for Windows. Armonk, NY: IBM Corp.) for factor analysis Mplus 7.1 (Muthén & Muthén. Released 2012. Los Angeles, CA: Muthén & Muthén) was used, and for calculation of the ordinal alpha R 3.1.1 for Windows (R Core Team. Released 2013. R: A language and environment for statistical computing. Vienna, Austria: R Foundation for Statistical Computing) was used.

## Results

### Patient characteristics

Mean age of the 223 elderly people included was 82.8 years (SD 4.7, range: 75 ‐ 100). Further demographic and health‐related characteristics are presented in Table 1.

**Table 1:** Respondent characteristics and results of the known‐groups validity test of the PAIEC (*n* = 223)

	*n* (%)	AIEC overall score	PAIEC subscales		
			Patient activation and contextual information	Goal setting and problem solving	Coordination and follow up
		Median scores (inter‐quartile range)			
Gender^a^					
Female	153 (69%)	26 (20–44)	10 (7–18)	8 (7–14)	7 (6–12)
Male	70 (31%)	33 (22–49)	12 (7–19)	10 (7–16)	10 (6–14)^b^
Age^a^					
≤82	126 (57%)	26 (20–44)	10 (7–17)	7 (7–14)	7 (6–13)
≥83	97 (43%)	33 (20–47)	12 (7–19)	10 (7–14)	8 (6–13)
Marital status^a^					
Married or long‐term relationship	109 (49%)	26 (20–44)	10 (7–16)	7 (7–14)	8 (6–13)
Widowed, divorced or single	114 (51%)	32 (21–46)	11 (7–19)	9 (7–14)	8 (6–13)
Education^c^					
Low^d^	127 (57%)	28 (20–45)	10 (7–18)	9 (7–15)	8 (6–13)
Moderate^d^	80 (36%)	32 (20–47)	12 (7–19)	9 (7–15)	9 (6–14)
High^d^	16 (7%)	27 (20–35)	11 (7–16)	9 (7–11)	6 (6–10)
Intervention profile^a^					
Frail	95 (43%)	28 (20–44)	10 (7–17)	7 (7–13)	8 (6–13)
Complex care needs	128 (57%)	30.5 (20–45.5)	11 (7–19)	10 (7–14.5)	8 (6–12.5)
Number of conditions^a^					
≤3	139 (62%)	24 (20–39)	9 (7–14)	7 (7–12)	7 (6–12)
≥4	84 (38%)	38 (25–49)^e^	15 (8–20)^e^	11 (7–16)^b^	11 (96–14)^e^
Number of medicines^a^					
≤3	62 (28%)	24 (20–37)	7.5 (7–14)	7 (7–11)	6 (6–10)
≥4	161 (72%)	32 (21–49)^b^	12 (7–19)^b^	10 (7–15)^b^	9 (7–13)^b^
Received home care^a^					
No	169 (76%)	25 (20–40)	9 (7–15)	7 (7–12)	7 (6–12)
Yes	54 (24%)	44 (32–52)^e^	17 (11–21)^e^	13 (9–19)^e^	11 (6–14)^b^

a Mann–Whitney test.

b Kruskal–Wallis test.

c Low: primary school, low vocational training or less; Moderate: secondary school or vocational training; High: higher professional education or university.

d Small effect size (*r *≥* *0.10 to <0.24).

e Moderate effect size (*r *≥* *0.24 to <0.37).

### Scale structure and reliability

The percentage of participants that used the response options ‘never’ varied from 7 to 32% per item and varied from 2 to 19% per item for the response option ‘always’. The percentage of participants that used the response options ‘does not apply’ varied from 31 to 51% per item; there were almost no missing values (0.1%).

The CFA of the *a priori* defined five‐factor model, as proposed by the developers of the PACIC,^12^ showed an insufficient fit for the data. The absolute indices showed poor fit [RMSEA: 0.086, 90% confidence interval (CI): (0.076–0.096) and WRMR 1.102]. The incremental fit indices showed acceptable results (CFI: 0.96 and TLI: 0.96). Considering the combination of the outcomes of the different goodness‐of‐fit statistics, the *a priori* defined five‐factor model was rejected.

Next, an EFA was performed. Only after excluding the item, ‘I was satisfied that my care and support was well organized’, did the EFA result in a solid three‐factor structure (Table 2). Factor loadings were > 0.40, items loaded on one factor only, and the estimated error variances were positive. In addition, model fit results were acceptable for absolute indices [RMSEA: 0.068, 90% CI (0.056–0.079), SRMR 0.042] and incremental fit indices (CFI: 0.98 and TLI: 0.97).

**Table 2:** Results of the exploratory factor analysis of the PAIEC a (*n* = 223)

Item		Factor		
		1	2	3
Patient activation and contextual information				
1	Asked for my ideas and expectations, when we made a care and support plan	**0.88**	0.00	−0.04
2	Given choices about care and support to think about	**0.82**	0.09	−0.01
3	Asked whether I had any problems with my medicines or their (side) effects	**1.08**	−0.30	0.00
4^b^	Asked whether I had any problems with my care and support, or what my experiences with either had been	**0.90**	−0.09	0.07
5	Given information on how to stay healthy or improve my health	**0.59**	0.14	0.21
12	Asked questions, either directly or on a survey, about my lifestyle (e.g., smoking, exercise, diet, etc.)	**0.41**	0.18	0.33
13	Sure that my healthcare professional thought about my values, beliefs, and traditions, when they recommended care and support to me	**0.58**	0.02	0.34
Goal setting and problem solving				
7	Explained how my own actions or behavior influenced my health	−0.04	**0.76**	0.25
8	Asked which goals I wished to achieve regarding my health	−0.36	**1.24**	0.00
9	Helped to set specific goals to deal with the consequences of ageing	−0.18	**1.07**	0.00
10	Given a copy of my care and support plan	0.01	**0.76**	0.11
14	Helped to make a care and support plan that I could carry out in my daily life	0.02	**0.57**	0.39
15	Helped to plan ahead so I could take care of myself in case my health declined or my situation worsened	0.02	**0.74**	0.17
16	Asked how the consequences of ageing affected my life	0.13	**0.53**	0.29
Coordination and follow up				
11	Contacted after a visit or after participating in a (group) activity to see how things were going	0.00	0.23	**0.61**
17	Encouraged to attend programs in the community that could help me	0.07	0.32	**0.51**
18	Referred to a healthcare professional (such as a physical therapist or social worker) or to a (group) activity	0.06	0.00	**0.79**
19	Explained why a visit to a healthcare professional or participation in an individual or group activity was important for me	−0.02	−0.23	**1.01**
20	Asked how my visits to (or by) healthcare professionals, or my participation in a (group) activity, were going	0.00	−0.15	**1.01**
21	Contacted after a visit or after participating in a (group) activity to see how things were going	0.07	0.20	**0.53**

The bold regressions coefficients indicate on which factor the item predominantly loaded.

a PACIC item 6 ‘I was satisfied that my care and support was well organized’ was excluded.

b Added PAIEC item.

The final version of PAIEC consists of 20 items divided into three scales. Based on the content of the items and keeping the original scale names in mind, scales were labelled as follows: ‘Patient activation and contextual information’, ‘Goal setting and problem‐solving’ and ‘Coordination and follow‐up’. The possible and observed scale scores, score distributions and internal consistencies are presented in Table 3. The internal consistencies of the PAIEC total and subscales were good, all above 0.8.

**Table 3:** Scale features of the PAIEC scales and subscales (*n* = 223)

	Items *K*	Possible scale scores	Observed scale scores	% Lowest score	% Highest score	Ordinal alpha
Overall score	20	20–100	20–94	28.7	0.0	0.97
Patient activation and contextual information	7	7–35	7–35	36.3	0.4	0.94
Goal setting and problem solving	7	7–35	7–35	46.6	0.6	0.96
Coordination and follow‐up	6	6–30	6–29	41.7	0.0	0.91

### Known‐groups validity

The results of the known‐groups validity tests are included in Table 1. As expected, the PAIEC scales were able to discriminate between groups of elderly people known to differ in terms of number of chronic conditions, number of medications and whether receiving home care or not. Patients with four or more chronic conditions, with four or more medications and people receiving home care experienced, a higher quality of integrated care than elderly people with fewer chronic conditions, fewer medications or without home care. All calculated effect sizes reflected a clinically relevant difference between subgroups. Differences were the strongest between groups of chronic conditions and receiving home care or not.

No differences were found for groups that differed in age, marital status, educational level and intervention profiles. However, there was a difference between men and women for the subscale ‘Coordination and follow‐up’. Women experienced the coordination of care and follow‐up to be of lower quality than men did.

### Divergent validity

As expected, regarding divergent validity, the correlations between the PAIEC variables and variables for life satisfaction, health status, complexity of care needs and frailty were weak (<0.3). This indicates that these measurement instruments assess different constructs than the PAIEC scales do (Table 4).

**Table 4:** Divergent validity of the PAIEC scales and subscales (*n* = 223)

	Median (interquartile range)	Overall score	Patient activation and contextual information	Goal setting and problem solving	Coordination and follow up
Life satisfaction					
Cantril's Ladder	7 (6–8)	−0.19^**^	−0.19^**^	−0.17^*^	−0.14^*^
Health status					
EQ5D‐5L index score	0.71 (0.59–0.80)	−0.22^**^	−0.25^**^	−0.21^**^	−0.14^*^
EQ5D‐5L VAS	60 (50–70)	−0.10	−0.12	−0.11	−0.02
Complexity of care needs					
IM‐E‐SA	15 (11–19)	0.19^**^	0.20^**^	0.22^**^	0.11
Frailty					
GFI	6 (4–8)	0.21^**^	0.21^**^	0.18^**^	0.14^*^

Spearman's rank order correlations (0.00–0.29 weak; 0.30–0.69 moderate; 0.70–1.00 strong).

^*^ *P* < 0.05; ^**^ *P* < 0.01.

## Discussion

The existing PACIC^12^, ^16^ was adapted to the PAIEC, a modified instrument wherein disease‐related concepts were converted into non‐disease‐specific concepts, the response option ‘does not apply’ was added, the item regarding medication and care and support was split into two items, and the one item concerning patient satisfaction was omitted. We found that the PAIEC had an acceptable three‐factor structure, demonstrated good internal consistencies and had reasonable construct validity.

Modifications made to the measurement instrument may contribute to the further development of the original PACIC and its derived questionnaires. For example, in the PAIEC the addition of the response option ‘does not apply’ led to almost no missing values. In previous PACIC validation studies, the percentage of missing values per item was up to 35% per item.^30^ The method commonly used for handling missing items in the PACIC is to replace them by mean scale scores. However, research by Drewes *et al*.^31^ showed that replacement of missing data by mean scale scores tends to artificially increase calculated PACIC scale scores for respondents with missing values, as compared to respondents without missing values. Although PAIEC scale sum scores will be relatively lower than PACIC scale mean scores, because the response option ‘does not apply’ and missing items (0.1% in this study) were recoded into ‘never’, the PAIEC scores will probably reflect care as experienced more realistically. In addition, using sum scores, an index score can be calculated, which facilitates total and subscale score comparisons.

Elderly participants selected for this study received intensive integrated care and support, but still frequently used the response options ‘not applicable’ and ‘never’. An explanation might be that these elderly participants had difficulty in remembering events that had occurred six months earlier.^32^ Alternatively, some elements of integrated care and support may not have been recognized as such by the participants, or these elements may actually not have been provided.^16^ Then again, considering that over 50% of the participants have a low educational level, it could also indicate that the questions were too difficult.^33^ Additional research is needed to gain insight into how respondents interpret the questions and choose response options.

Analysis of the factor structure resulted in the rejection of the *a priori defined* five‐factor model in favour of a three‐factor structure in the PAIEC. In many PACIC validation papers, difficulties have been reported as well, confirming the *a priori defined* five‐factor structure, while various new factor structures have been reported.^30^, ^31^, ^32^, ^33^, ^34^, ^35^, ^36^, ^37^, ^38^ We suggest that this might be related to the item concerning patient *satisfaction* (‘I was satisfied that my care and support was well organized’). As established in previous research, questions regarding *satisfaction with care* assess a different construct (and also lead to a higher valuation) than questions regarding *care as experienced*.^39^, ^40^, ^41^ Because the measurement instrument was explicitly developed to assess the receipt of CCM‐based care and support and not patient satisfaction, this item was omitted from the PAIEC. After deleting this item, a satisfying and clear three‐factor structure was found. Maybe this redundant item explains the difficulties in finding an unambiguous factor structure in the aforementioned PACIC validation studies.

Acceptable results for the divergent and known‐groups validity tests were found. However, women reported lower quality of care than men did, especially regarding coordination of care and follow‐up. Results of PACIC validation studies^12^, ^34^ showed differences between gender as well. One explanation might be that women more frequently provided informal care and therefore were more critical. Further research is needed to gain further insight into the origin of this gender difference.

Strengths of this study were the use of a representative sample of elderly people receiving integrated care and support, and the application of robust nonparametric tests for the assessment of the psychometric properties. Some potential limitations could be addresses as well. For this study, a sample of elderly people whom were living in a rural area, were 75 years and older and had a relatively low SES, was selected. The rather specific nature of this sample may have affected the outcomes of this study, potentially leading to bias.^42^ Finally, due to practical reasons not all statistical methods could be applied, for example convergent and test–retest validity.^43^

Further research is needed to confirm the PAIECs’ validity with use of additional criteria, and among other populations, that is other age‐groups and ‘robust’ elderly persons. It is recommended to assess the PAIEC's cross‐cultural validity before its use in other countries or in other ethnic populations.^43^ Finally, some of the modifications made in this questionnaire could be applied to the original PACIC for chronic conditions and its derived questionnaires.

The PAIEC can have major implications for policy and practice. It enables all stakeholders to incorporate the perspectives of elderly persons in quality‐of‐care evaluations and improvements, the co‐creation of integrated care interventions, and further integration of services and funding within the health‐care system.^44^ However, when interpreting the results of the PAIEC it is of importance to take into account the degree of integration of the, still evolving, services as offered, because this probably will influence the PAIEC scores.^45^ To be able to estimate the degree of integration, it is important to incooperate, for example, the perspectives of professionals and managers^46^ using the closely related Assessment of Chronic Illness Care questionnaire,^47^ or to apply other measurement instruments.^48^

In conclusion, we designed and validated the PAIEC, an adapted version of the PACIC wherein items and response options were modified to fit the population of elderly people while the essence of the measurement instrument remained unaffected. The PAIEC seems to have a considerable potential as a suitable, reliable and valid measurement instrument for assessing the quality of integrated care from the perspective of elderly people.

## Funding

Funded by the Netherlands Organization for Health Research and Development (ZonMw) file number 314010201 and the Dutch Healthcare Authority (NZA) file number 300‐1021. The Netherlands National Trial Register: NTR3039

## Conflict of interests

Our manuscript has not been published and is not under consideration for publication elsewhere. The authors declare that they have no competing interests, including specific financial interests and relationships, and no affiliations relevant to the subject. The manuscript has been prepared in accordance with the style of the journal and has been approved by all authors. All named authors have contributed substantially to the conception and design, or have drafted or revised the article critically for important intellectual content, and have agreed to submission of this manuscript.

## Supporting information


**Appendix S1.** Patient Assessment of Integrated Elderly Care.
