# Supplementary material for: Development and psychometric evaluation of a measure to evaluate the quality of integrated care: the Patient Assessment of Integrated Elderly Care
Source: Health Expect. 2015 Jul 31;19(4):962–72. doi: 10.1111/hex.12391 (PMC5042070; doi:10.1111/hex.12391)
Supplement: Supplementary file 1 — Appendix S1. Patient Assessment of Integrated Elderly Care. [file HEX-19-962-s001.docx]

# Appendix A Patient Assessment of Integrated Elderly Care

| **Over the past six months, when I received care and support for health issues related to ageing or my chronic condition(s), I was…** | *None of the time* | *A little of the time* | *Some of the time* | *Most of the time* | *Always* | *Does not apply* |
| --- | --- | --- | --- | --- | --- | --- |
| - - - 1. Asked for my ideas and expectations, when we made a care and support plan | □ | □ | □ | □ | □ | □ |
| - - - 1. Given choices about care and support to think about | □ | □ | □ | □ | □ | □ |
| - - - 1. Asked whether I had any problems with my medicines or their (side) effects | □ | □ | □ | □ | □ | □ |
| - - - 1. Asked whether I had any problems with my care and support or what my experiences with either had been | □ | □ | □ | □ | □ | □ |
| - - - 1. Given information on how to stay healthy or improve my health | □ | □ | □ | □ | □ | □ |
| - - - 1. Explained how my own actions or behavior influenced my health | □ | □ | □ | □ | □ | □ |
| - - - 1. Asked which goals I wished to achieve regarding my health | □ | □ | □ | □ | □ | □ |
| - - - 1. Helped to set specific goals in dealing with the consequences of ageing | □ | □ | □ | □ | □ | □ |
| - - - 1. Given a copy of my care and support plan | □ | □ | □ | □ | □ | □ |
| - - - 1. Encouraged to take a course, participate in a group, or undertake activities to help me cope with the consequences of ageing | □ | □ | □ | □ | □ | □ |
| - - - 1. Asked questions, either directly or on a survey, about my lifestyle (e.g., smoking, exercise, diet, etc.) | □ | □ | □ | □ | □ | □ |
| - - - 1. Sure that my healthcare professional had thought about my values, beliefs, and traditions, when they recommended care and support to me | □ | □ | □ | □ | □ | □ |
| - - - 1. Helped to make a care and support plan that I could carry out in my daily life | □ | □ | □ | □ | □ | □ |
| - - - 1. Helped to plan ahead so I could take care of myself in case my health declined or my situation worsened | □ | □ | □ | □ | □ | □ |
| - - - 1. Asked how the consequences of ageing affected my life | □ | □ | □ | □ | □ | □ |
| - - - 1. Contacted after a visit or after participating in a (group) activity to see how things were going | □ | □ | □ | □ | □ | □ |
| - - - 1. Encouraged to attend programs in the community that could help me | □ | □ | □ | □ | □ | □ |
| - - - 1. Referred to a healthcare professional (such as a physical therapist or social worker) or to a (group) activity | □ | □ | □ | □ | □ | □ |
| - - - 1. Explained why a visit to a healthcare professional or participation in an individual or group activity was important for me | □ | □ | □ | □ | □ | □ |
| - - - 1. Asked how my visits to (or by) health-care professionals, or my participation in a (group) activity, were going | □ | □ | □ | □ | □ | □ |
